# Supplementary material for: Optimized cryo-FIB milling strategy to generate thin, minimally damaged biological lamellae
Source: bioRxiv. 2026 Jul 25:2026.07.21.739890. Preprint. [Version 1] doi: 10.64898/2026.07.21.739890 (PMC13419515; doi:10.64898/2026.07.21.739890)
Supplement: 1 [file NIHPP2026.07.21.739890v1-supplement-1.pdf]

bioRxiv preprint doi: https://doi.org/10.64898/2026.07.21.739890; this version posted July 25, 2026. The copyright holder for this preprint (which was not certified by peer review) is the author/funder, who has granted bioRxiv a license to display the preprint in perpetuity. It is made available under a CC-BY 4.0 International license.

728 # Supplementary Information

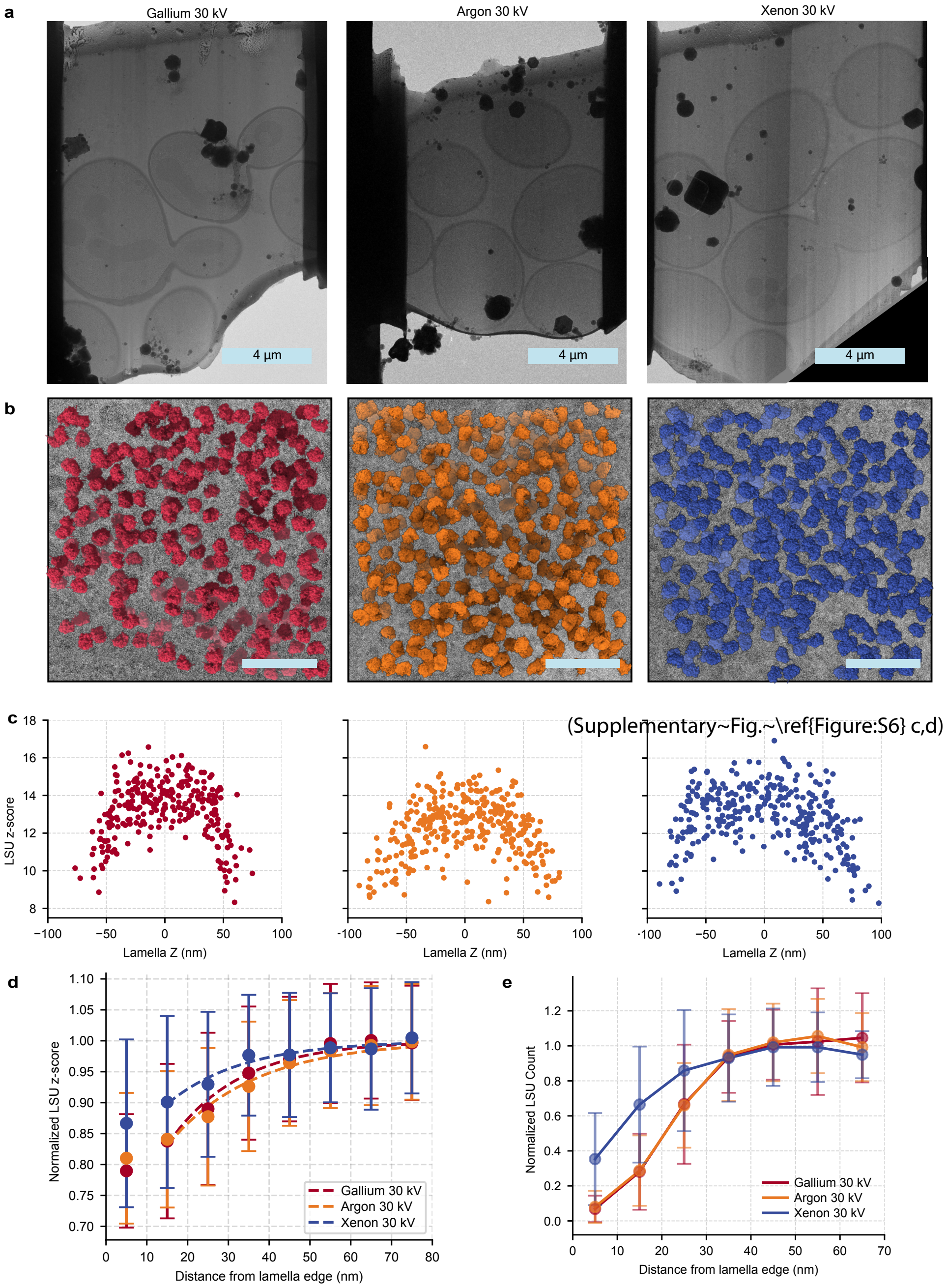

Figure S1. Xenon reduces FIB Damage at 30 kV. (a) TEM images of example lamellae milled with gallium, argon and xenon at 30 kV. Scale bar: 4 µm **(b)** Example images from lamellae milled with each ion overlaid with ribosomal LSUs in the locations and orientations identified with 2DTM. Scale bar: 50 nm. **(c)** Scatterplot of the mean 2DTM z-scores of LSUs as a function of distance from lamella edge in 10 nm bins normalized to undamaged bins in the center of the lamella from the same images of argon-milled (yellow), xenon-milled (blue) or gallium-milled (red) lamellae. **(d)** Scatterplot showing the normalized 2DTM z-scores of multiple images, plotting SNR as a function of distance from lamella edge. All z-scores were normalized to the undamaged bins > 70 nm from the lamella edge from the same image. Curves represent the fits of the exponential decay function. Error bars indicate the standard deviation. **(e)** Number of LSUs identified in depth bins from lamella surface from the same subset of images from part (d). Normalized to full undamaged bin counts at > 70 nm from the lamella edge in individual images. Error bars indicate the standard deviation.

bioRxiv preprint doi: https://doi.org/10.64898/2026.07.21.739890; this version posted July 25, 2026. The copyright holder for this preprint (which was not certified by peer review) is the author/funder, who has granted bioRxiv a license to display the preprint in perpetuity. It is made available under a CC-BY 4.0 International license.

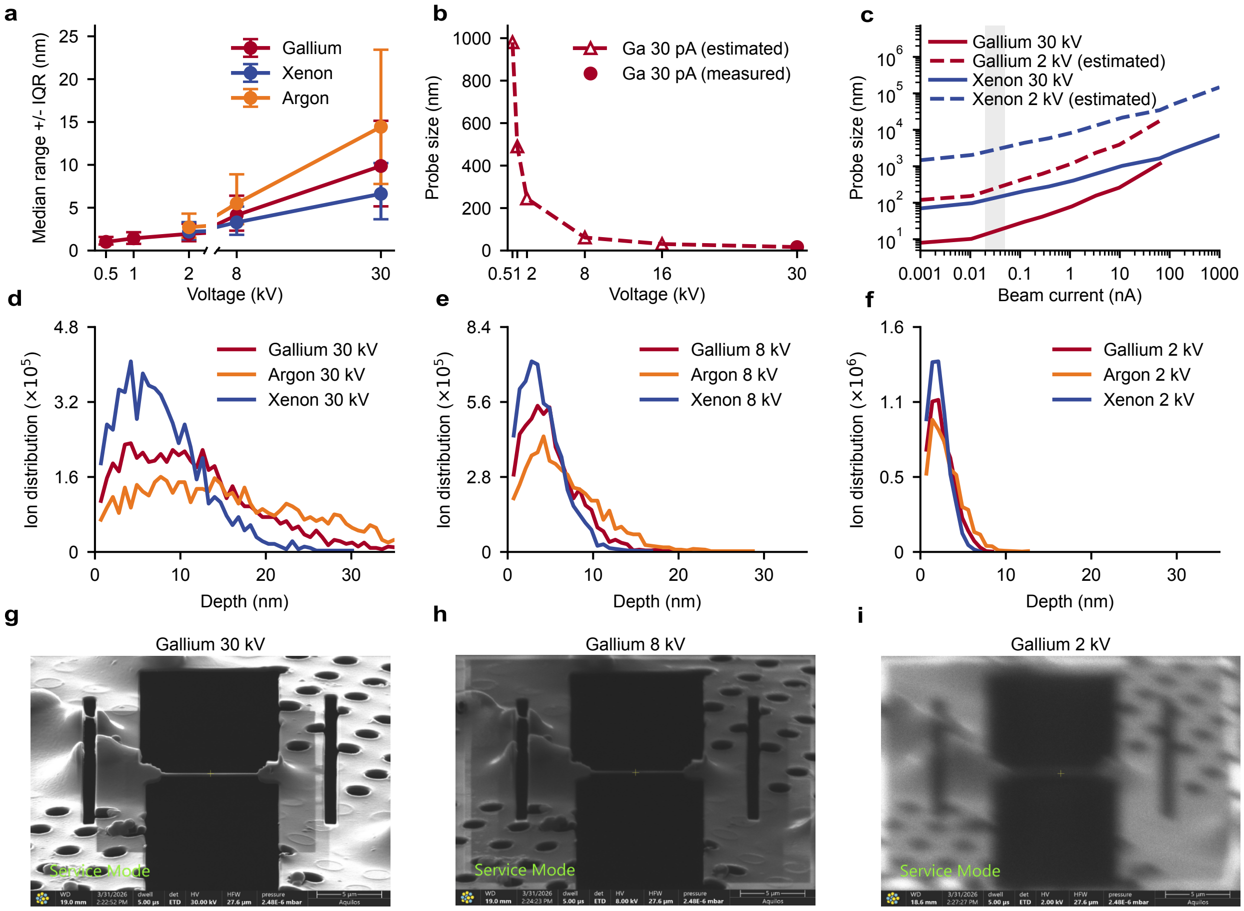

Figure S2. Justifying the selection of gallium for low-voltage milling. (a) SRIM simulated median range for gallium, argon and xenon at voltages from 0.5 kV to 30 kV. Error bars indicate the IQR. **(b)** Probe size of Gallium at voltages from 0.5 kV to 30 kV. Value for the 30 kV probe was experimentally determined[1] and lower voltage probe sizes were predicted using $d_c \propto \frac{\Delta U}{U}$, where d_c is the probe diameter, ΔU is the energy spread of the beam, and U is the accelerating voltage[2]. **(c)** Probe size for Gallium and Xenon across beam currents, produced as in (b). Grey highlighted region indicates common polishing currents. **(d,e,g)** SRIM simulation for ion distribution of Argon, Gallium Xenon at 30 kV, 8 kV and 2 kV in "frozen yeast" (see methods). **(g,h,i)** FIB images at 30 kV, 8 kV, and 2 kV showing image blurring due to increased probe size at low voltage.

bioRxiv preprint doi: https://doi.org/10.64898/2026.07.21.739890; this version posted July 25, 2026. The copyright holder for this preprint (which was not certified by peer review) is the author/funder, who has granted bioRxiv a license to display the preprint in perpetuity. It is made available under a CC-BY 4.0 International license.

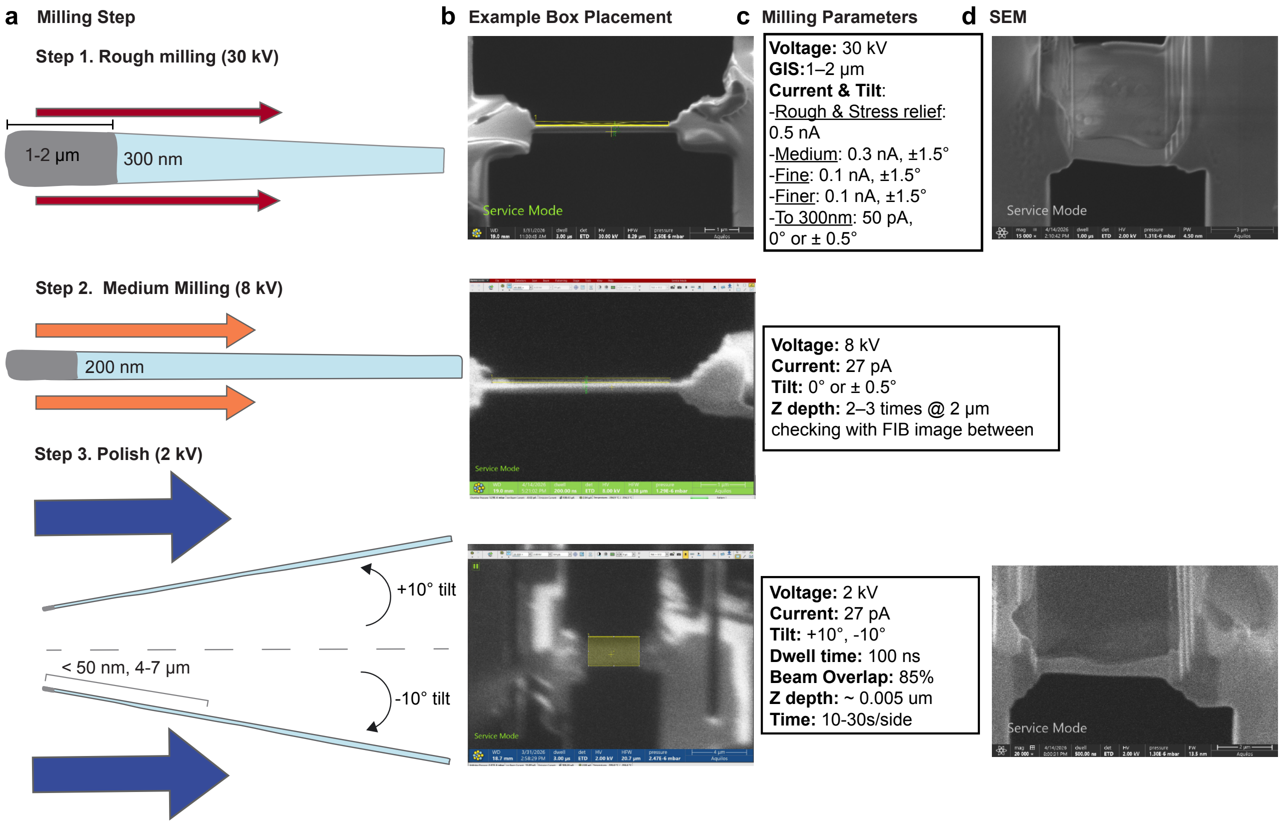

Figure S3. Steps, example pattern placements, and milling parameters for Nilas. (a) Schematic of the three voltages steps of the Nilas method. **(b)** Example milling pattern placement for each Nilas step as seen in the FIB image. Cleaning cross section patterns are placed as typical for 30 kV and without overlaying the visible area of the lamellae due to the increased beam size at 8 kV. For 2 kV a rectangle pattern is drawn broadly across the lamella surface. **(c)** Milling parameters used at each Nilas step. **(d)** SEM images of lamellae after completion of the corresponding step, SEM contrast changes as lamella is thinned.

bioRxiv preprint doi: https://doi.org/10.64898/2026.07.21.739890; this version posted July 25, 2026. The copyright holder for this preprint (which was not certified by peer review) is the author/funder, who has granted bioRxiv a license to display the preprint in perpetuity. It is made available under a CC-BY 4.0 International license.

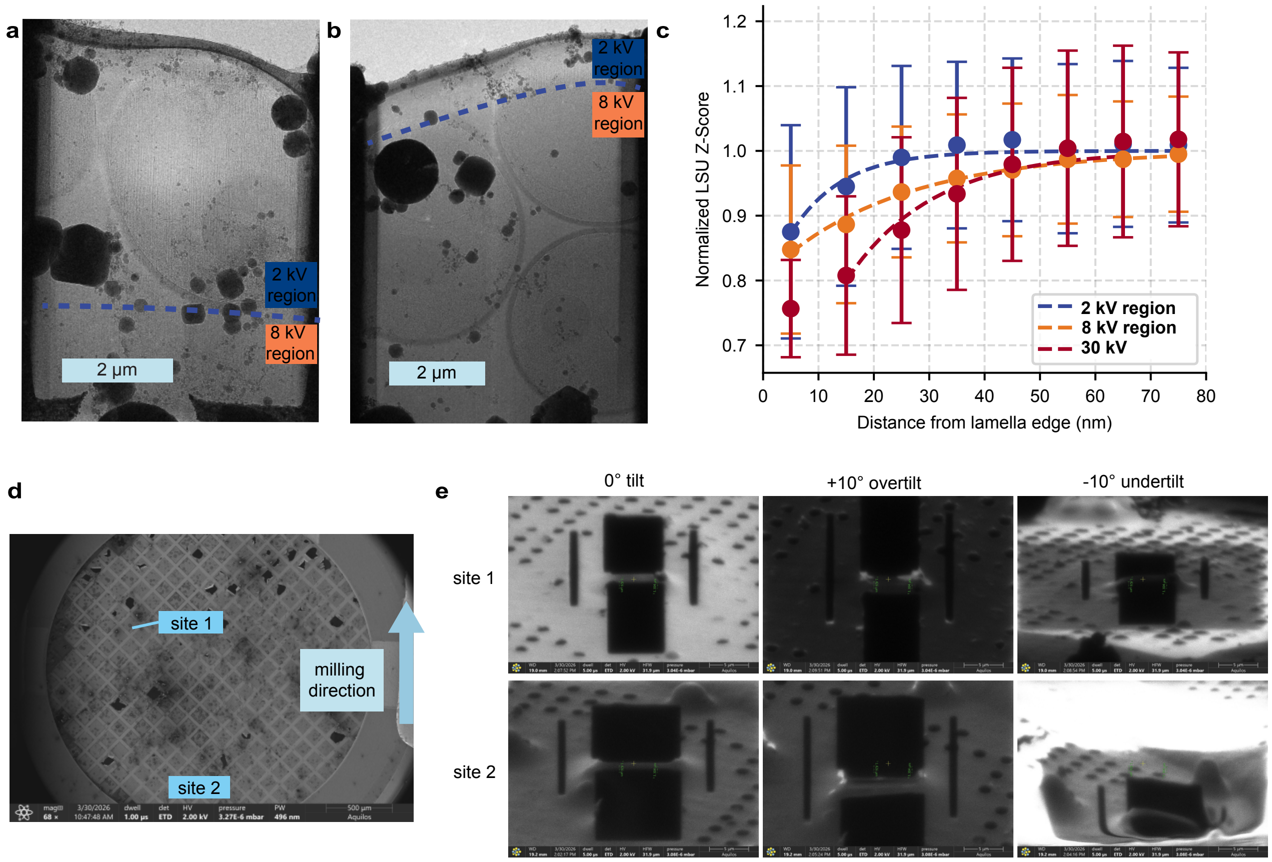

Figure S4. A ±10 degree tilt is required to extend 2 kV damage region. (a) Example TEM image of a lamella polished at 2 kV with +10 degree tilt (relative to initial milling angle). Scale bar: 2 µm. **(b)** Example TEM image of a lamella polished at 2 kV with a +4 degree tilt. Scale bar: 2 µm. **(c)** Scatterplot of the mean 2DTM z-scores of LSUs binned by distance to lamella edge normalized to undamaged bins in the center of images in gallium- milled at 30 kV (red), gallium- milled at 8 kV (orange) or gallium-milled at 2 kV (red) lamellae regions. Curves represent fits of the exponential decay function. Error bars indicate the standard deviation. (**d**) An SEM image showing the overview of a grid with two milling sites: site 1 (nearer to the FIB source) and site 2 (further from the FIB source). The FIB source is towards the bottom of the image. **(e)** Images depicting the 2 kV FIB image warping during the 10 degree under-tilt as a result of the ion beam illuminating the clip ring.

bioRxiv preprint doi: https://doi.org/10.64898/2026.07.21.739890; this version posted July 25, 2026. The copyright holder for this preprint (which was not certified by peer review) is the author/funder, who has granted bioRxiv a license to display the preprint in perpetuity. It is made available under a CC-BY 4.0 International license.

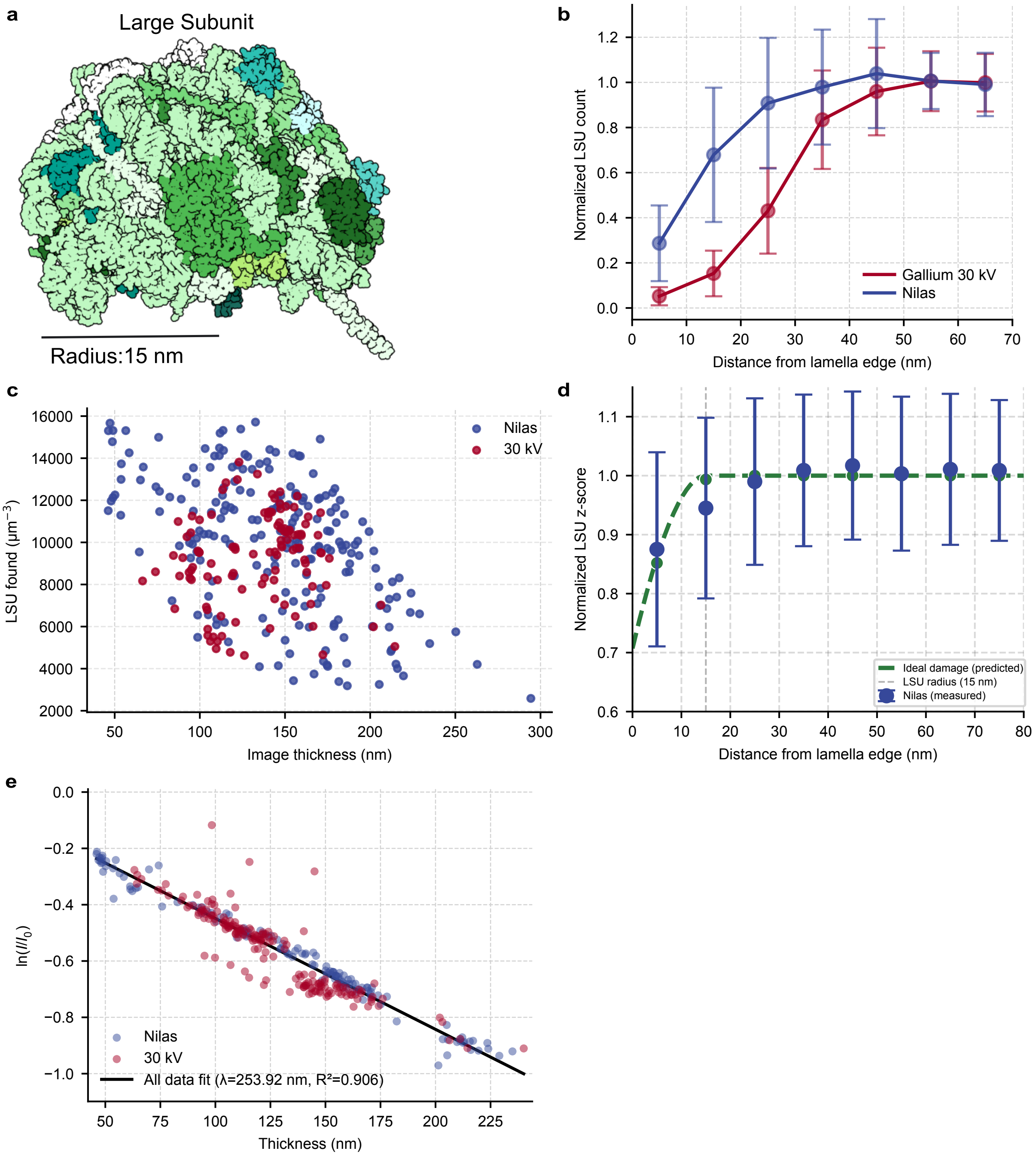

Figure S5. Measuring FIB-damage with 2DTM of the LSU. (a) A model of the LSU of the ribosome (PDB:6Q8Y) indicating its radius of 15 nm. **(b)** The number of LSUs identified in 10 nm bins relative to the lamella surface normalized to the number of significant LSU detections in an undamaged bin. Error bars indicate the standard deviation. **(c)** The concentration of LSUs detected per image in Nilas and 30 kV images as a function of thicknesses. **(d)** The predicted ideal damage curve (green) is a result of the effect of LSU ablation on the 2DTM z-score ignoring the effects of sub-surface damage. Nilas-milled lamellae damage curve (blue) from (Fig. 2e) for comparison. **(e)** The CTFFIND5 determined thickness plotted against the natural log of image intensity over vacuum intensity. The plot demonstrates a Beer-Lambert law relationship between thickness and transmitted signal ln(I/I_0).

bioRxiv preprint doi: https://doi.org/10.64898/2026.07.21.739890; this version posted July 25, 2026. The copyright holder for this preprint (which was not certified by peer review) is the author/funder, who has granted bioRxiv a license to display the preprint in perpetuity. It is made available under a CC-BY 4.0 International license.

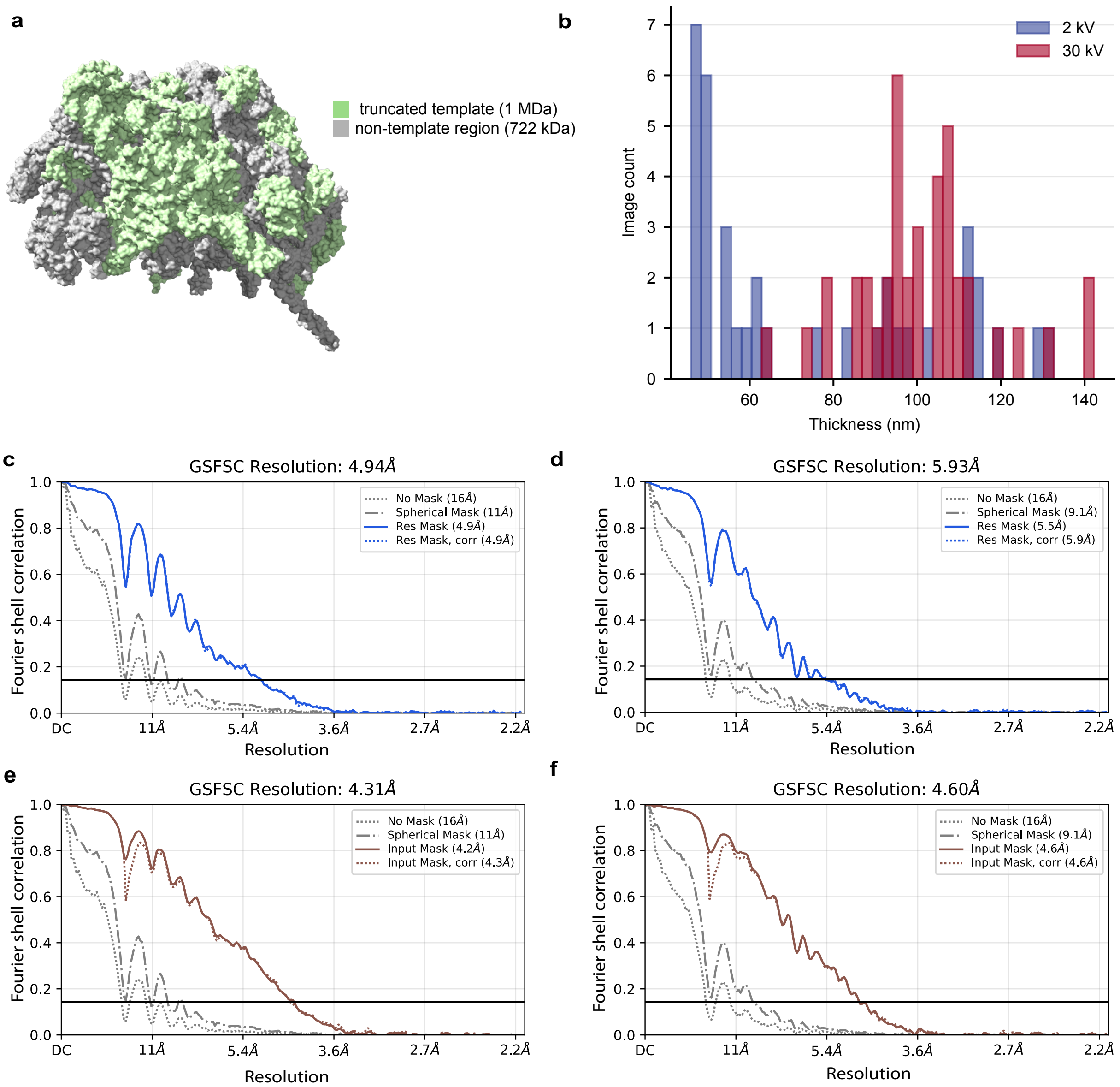

Figure S6. Baited reconstructions using 1 MDa LSU fragment. **(a)** LSU truncated 1 MDa template in grey and non-template region (722 kDA) in green. **(b)** Histogram of CTFFIND5 estimated thickness for images used in the reconstructions in (Fig. 3a) for each condition. **(c)** Fourier shell correlation (FSC) curve for the Nilas homogeneous reconstruction in (Fig. 3a). FSC oscillation is due to narrow defocus range of 2DTM- identified particles. Resolution determined at FSC value of 0.143 (black line). **(d)** FSC curve for the 30 kV LSU homogeneous reconstruction in (Fig. 3a) FSC oscillation is due to narrow defocus range of 2DTM-identified particles. Resolution determined at FSC value of 0.143 (black line). **(e)** FSC curve for the LSU region omitted from the 2DTM template from the homogeneous reconstruction using particles identified in 30 kV-milled lamellae in (Fig. 3c). **(f)** As for (e) using particles identified in Nilas-milled lamellae.

bioRxiv preprint doi: https://doi.org/10.64898/2026.07.21.739890; this version posted July 25, 2026. The copyright holder for this preprint (which was not certified by peer review) is the author/funder, who has granted bioRxiv a license to display the preprint in perpetuity. It is made available under a CC-BY 4.0 International license.

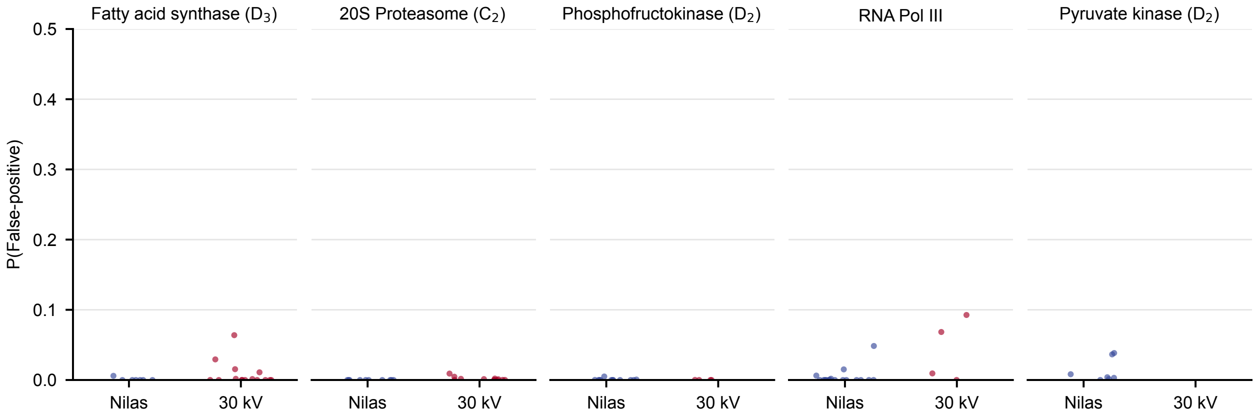

Figure S7. The probability of false positive detections with 2DTM on non-ribosomes. The false positive probability of 2DTM peaks based on their z-scores as calculated by the Gaussian noise background model for 2DTM peaks from (Fig 5).

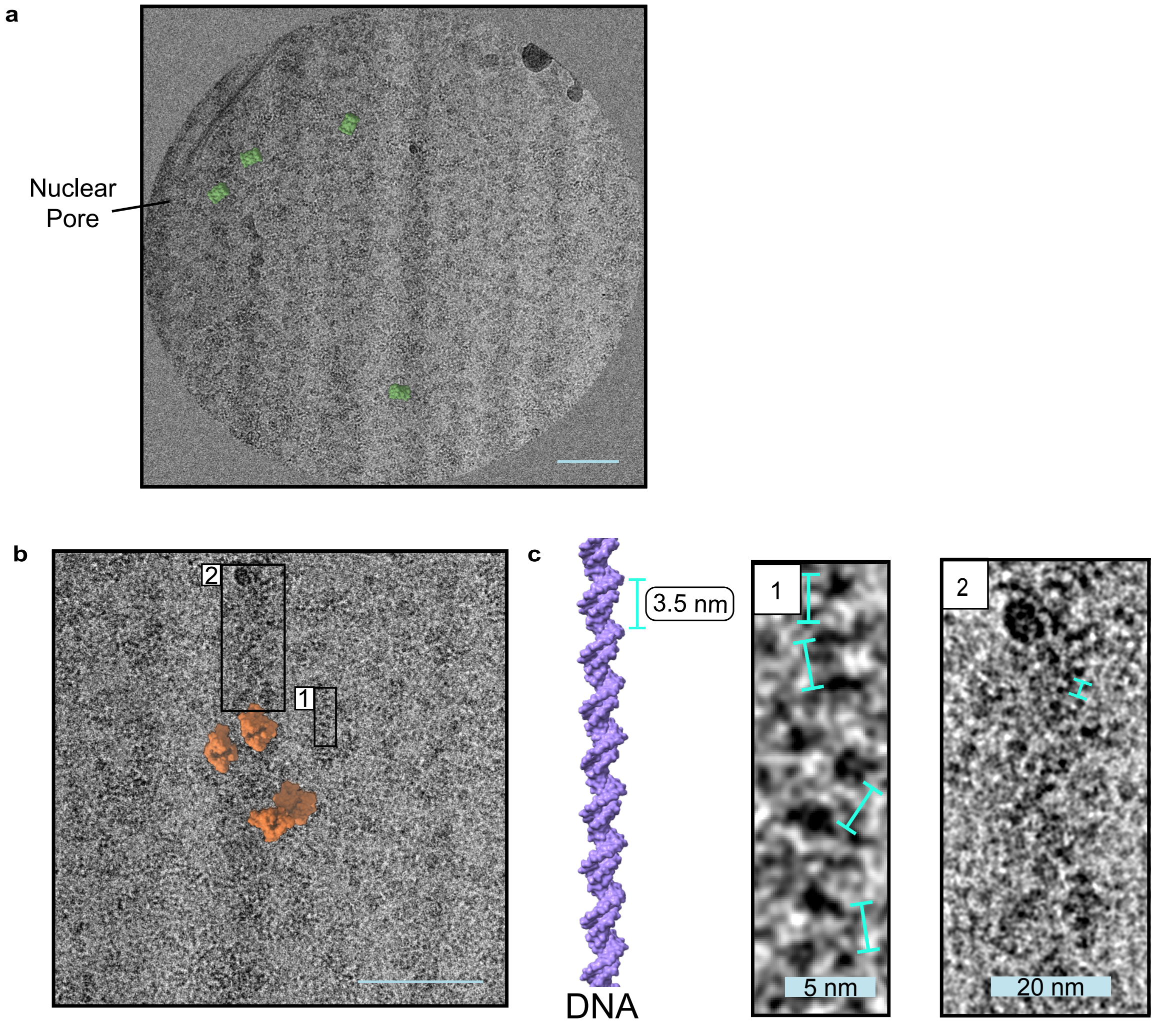

Figure S8. Observations enabled by thin lamellae with Nilas. (**a**) Overlay of the proteasome template for locations and orientations identified by 2DTM on an image region with nuclear membrane and nuclear pore visible. Scale bar: 50 nm. (**b**) Overlay of RNA polymerase III template for locations and orientations identified by 2DTM on an image region with RNA polymerase III in orange. Scale bar: 50 nm. (**c**) A DNA model (PDB: 4BNA) scaled to the same magnification as (1). Inset 1: from part (b) showing alternating contrast caused by DNA. Teal markers represent 3.5 nm which is consistent with the pitch of DNA. Scale bar: 5 nm. Inset 2: from part (b), suspected DNA super-coiling. Scale bar: 20 nm.

bioRxiv preprint doi: https://doi.org/10.64898/2026.07.21.739890; this version posted July 25, 2026. The copyright holder for this preprint (which was not certified by peer review) is the author/funder, who has granted bioRxiv a license to display the preprint in perpetuity. It is made available under a CC-BY 4.0 International license.

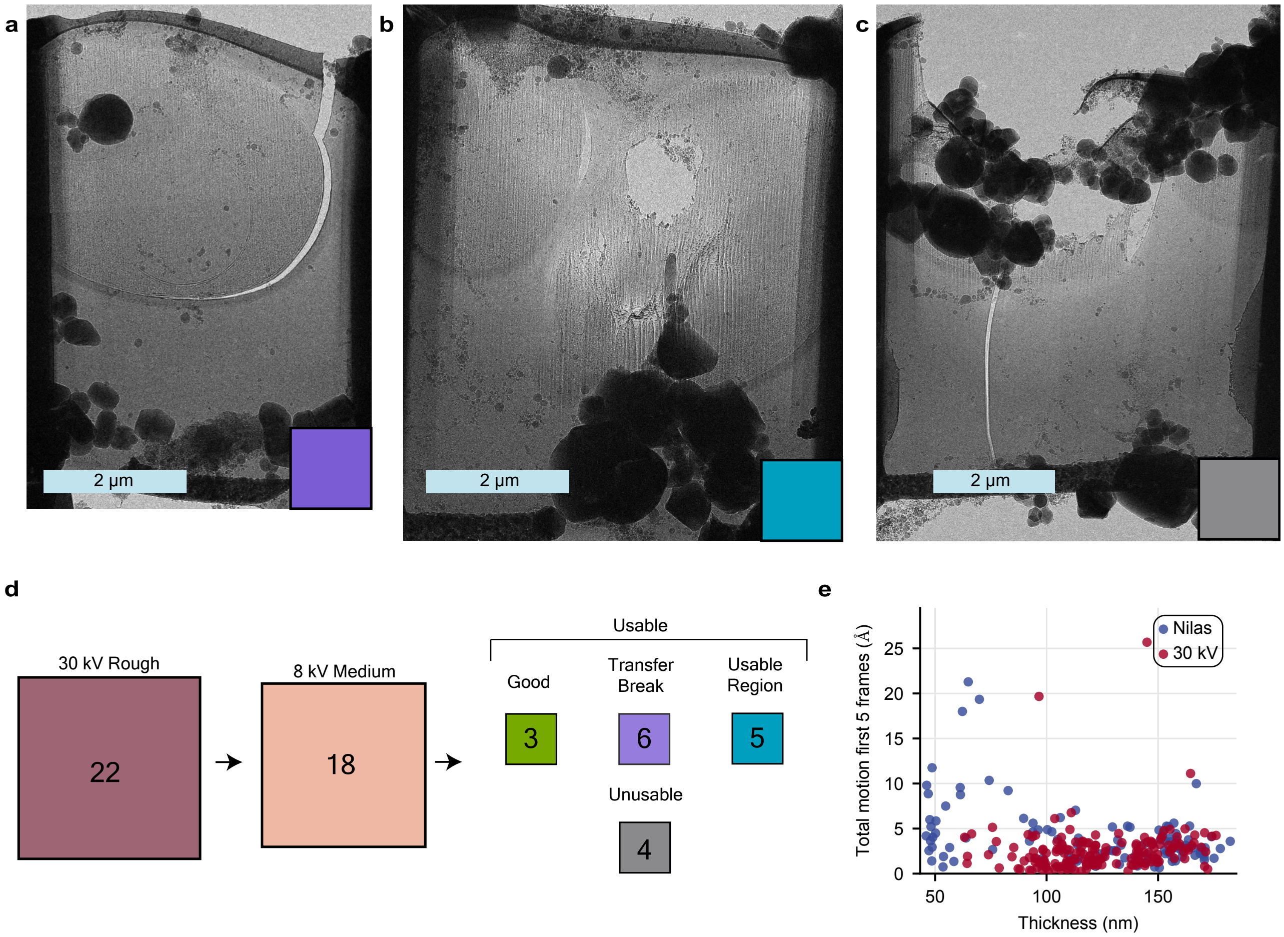

Figure S9. The throughput and stability of Nilas lamellae. TEM images of an example lamella that **(a)** broke at the cell membrane and cell wall boundary between the FIB and TEM position **(b)** was over-milled with 2 kV resulting in excessive curtaining and a hole in a less dense region and **(c)** had the organo-platinum layer milled away resulting in breakage. Scale bar: 2 µm. **(d)** The throughput of Nilas. 22 Nilas lamellae were originally attempted and 18 were successfully milled with 8 kV. Of those 18, 3 were excellent, 6 broke during transfer between the FIB and TEM (a), 5 had some usable regions but contained holes or cracks (b) and 4 were completely unusable (c). **(e)** Summed inter-frame global motion of the first 5 frames (5 $e^{-}/Å^{2}$) for Nilas and 30 kV-milled images at various thicknesses.

bioRxiv preprint doi: https://doi.org/10.64898/2026.07.21.739890; this version posted July 25, 2026. The copyright holder for this preprint (which was not certified by peer review) is the author/funder, who has granted bioRxiv a license to display the preprint in perpetuity. It is made available under a CC-BY 4.0 International license.

1. Vitale, S. M. & Sugar, J. D. Using xe plasma fib for high-quality tem sample preparation. *Microscopy and Microanalysis* **28**, 646–658 (2022).
2. McClelland, J. J. *et al.* Bright focused ion beam sources based on laser-cooled atoms. *Applied Physics Reviews* **3**, 011302 (2016).
